# Adaptive Detection in Real-Time Gait Analysis through the Dynamic Gait Event Identifier

**DOI:** 10.3390/bioengineering11080806

**Published:** 2024-08-08

**Authors:** Yifan Liu, Xing Liu, Qianhui Zhu, Yuan Chen, Yifei Yang, Haoyu Xie, Yichen Wang, Xingjun Wang

**Affiliations:** 1 Shenzhen International Graduate School, Tsinghua University, Shenzhen 518055, China; liuxing21@tsinghua.org.cn (X.L.); zhuqh23@mails.tsinghua.edu.cn (Q.Z.); chenyuan23@mails.tsinghua.edu.cn (Y.C.); yangyf21@mails.tsinghua.edu.cn (Y.Y.); wang-yc22@mails.tsinghua.edu.cn (Y.W.); 2 Huawei Cloud, Shanghai 200121, China; 3 College of Arts and Sciences, The University of North Carolina at Chapel Hill, Chapel Hill, NC 27514, USA; haox@ad.unc.edu

**Keywords:** embedded algorithm, gait event detection, dynamic feature extraction, IMU signals

## Abstract

The Dynamic Gait Event Identifier (DGEI) introduces a pioneering approach for real-time gait event detection that seamlessly aligns with the needs of embedded system design and optimization. DGEI creates a new standard for gait analysis by combining software and hardware co-design with real-time data analysis, using a combination of first-order difference functions and sliding window techniques. The method is specifically designed to accurately separate and analyze key gait events such as heel strike (HS), toe-off (TO), walking start (WS), and walking pause (WP) from a continuous stream of inertial measurement unit (IMU) signals. The core innovation of DGEI is the application of its dynamic feature extraction strategies, including first-order differential integration with positive/negative windows, weighted sleep time analysis, and adaptive thresholding, which together improve its accuracy in gait segmentation. The experimental results show that the accuracy rate of HS event detection is 97.82%, and the accuracy rate of TO event detection is 99.03%, which is suitable for embedded systems. Validation on a comprehensive dataset of 1550 gait instances shows that DGEI achieves near-perfect alignment with human annotations, with a difference of less than one frame in pulse onset times in 99.2% of the cases.

## 1. Introduction

In the evolving landscape of embedded systems, the integration of hardware and software components to achieve highly efficient, real-time processing capabilities is paramount. The advent of sophisticated methodologies that can seamlessly analyze continuous data streams [1,2] in real time not only pushes the boundaries of what embedded systems can achieve but also opens new avenues for application-specific designs [3]. Among these, real-time detection and analysis of human gait events holds particular significance due to its wide range of applications, from medical diagnostics [4] and rehabilitation [5] to the design of adaptive and reconfigurable systems [6]. This paper presents the Dynamic Gait Event Identifier (DGEI), a novel approach designed to meet these challenges head-on, embodying the principles of hardware/software co-design and system synthesis at its core.

The demand for real-time gait analysis in embedded systems is driven by the need for precise, reliable, and efficient detection of specific gait events such as heel strike (HS), toe-off (TO), walking start (WS), and walking pause (WP) [7,8]. These events are critical markers in understanding human locomotion [9] and are indispensable in applications that require adaptive responses based on gait patterns. Heel strike (HS) occurs when the heel first touches the ground, marking the start of the stance phase. Toe-off (TO) is the moment when the toes leave the ground, transitioning from the stance phase to the swing phase. Walking start (WS) refers to the initiation of walking motion, characterized by a distinct change in gait dynamics. Walking pauses (WPs) are brief periods of rest during which the individual stops walking, often occurring between steps.

However, the inherent complexity of accurately capturing these events in real time, especially within the constrained environment of embedded systems [10], poses a significant challenge. Firstly, these systems need to process and analyze sensor data in real time [11], which requires algorithms with low latency and efficient computation [12]. Secondly, since the gait patterns of different individuals are significantly different [13], the algorithm must have a high adaptability and generalization ability. Moreover, embedded systems typically operate under a variety of environmental conditions [14], including different ground conditions and activity intensities, which further increases the complexity of algorithm design. Finally, users also have high expectations for the comfort and wearability of devices [15], which requires the design of sensors and algorithms to be as light and unobtrusive as possible. This necessitates a solution that not only leverages the latest in data processing techniques but is also embedded within the system-level design to ensure seamless integration and operation.

In the sphere of wearable device and adaptive support systems, accurately detecting the moments of “gait initiation” and “gait termination” is crucial for interpreting user intentions [16], serving as key indicators for engaging and disengaging assistive modalities. “Gait initiation” signifies the commencement of movement, marking a critical point for enabling supportive features that harmonize with the user’s intended motion. Conversely, “gait termination” indicates the cessation of movement, requiring the system to reduce or halt movement [17], thus ensuring a smooth transition to a stationary state. These moments are integral to the design and operation of advanced systems [18,19] focused on mobility assistance, playing a pivotal role in optimizing user interaction and enhancing the overall experience [20]. Accurately identifying these events not only improves the functionality of such systems but also significantly impacts user safety and comfort [21].

Moreover, the events of toe-off (TO) and heel strike (HS) are critical milestones within the gait cycle [22,23], having profound implications for the design and functionality of mobility assistance systems. TO marks the transition to the swing phase of walking, as the foot leaves the ground, playing a crucial role in ensuring the body’s stabilization and readiness for the next step. On the other hand, HS signifies the beginning of the stance phase [24], with the heel’s first contact with the ground providing essential stability and mitigating impact forces during walking. These moments are pivotal for the timely activation of supportive mechanisms, which can offer propulsive assistance and enhance the walking efficiency of users. Understanding and accurately identifying TO and HS events are essential for optimizing the performance of systems designed to improve mobility and ensure user safety.

Therefore, the precise identification and responsive adaptation to these four essential gait events—heel strike (HS), toe-off (TO), walking start (WS), and walking pause (WP) [25]—from continuous data streams are paramount for ensuring the synchronization of assistive systems with the user’s natural gait rhythm. Achieving this harmonization is critical for enhancing the effectiveness and comfort of mobility support, as well as safeguarding the user during its operation.

Traditional methods of gait analysis often struggle to discern these subtle but vital differences, particularly when considering the variability in gait patterns and the influence of different environmental factors. Variations in the amplitude and frequency of foot movements, which fluctuate with changes in step frequency and speed during activities like walking or running, introduce additional complexity to the task of gait event detection, necessitating advanced analytical techniques to achieve reliable accuracy.

Additionally, the accurate discernment of peaks within gait data poses a significant challenge. In real-world gait analysis, once a peak associated with toe-off is identified, the algorithm must be cautious not to erroneously interpret closely spaced peaks in a brief period, as consecutive toe-off events are improbable within a typical gait cycle. This necessitates a mechanism to momentarily “suspend” the algorithm, preventing the misidentification of proximate peaks, and thus, reducing false alarms stemming from noise.

Another persistent hurdle is the precise identification and adjustment to shifts in gait curve amplitudes across different scenarios. Traditional gait analysis often relies on fixed thresholds for detecting events like toe-off or heel strike. This method typically lacks the adaptability required to manage changes under varying gait conditions, especially when shifts in gait speed and pattern occur, such as transitioning from a slow walk to a sprint or navigating varied terrains, where fixed thresholds might not effectively capture all pivotal gait events.

Moreover, gait curve variations are subject to the impacts of individual disparities, activity intensity, and environmental elements, posing intertwined challenges that necessitate a holistic approach. Activities of different intensities, such as high-speed or intense movements, often result in greater amplitudes and jitters with elevated event frequencies, while slower or milder activities yield comparatively lower amplitudes and frequencies. Such amplitude and frequency discrepancies across various activity intensities can potentially lead to inaccuracies in gait event detection. Thus, a method that is both flexible and all-encompassing is required to navigate these intricate fluctuations and challenges.

Confronting these challenges head-on, our research introduces a groundbreaking approach: the DGEI, a high-precision, multi-event real-time gait detection methodology optimized for complex settings. This technique harnesses Euler angle data from the lower limbs of assistive systems, significantly enhancing the detection accuracy of pivotal gait events. The DGEI stands out for its remarkable sensitivity and adaptability to the nuances of diverse gait patterns, particularly in dynamic and variable environments.

The primary contributions of this paper are as follows:

- High-precision multi-event real-time gait detection: We present a novel gait detection method suitable for complex settings, characterized by high precision and the ability to handle multiple events in real time. This method not only elevates the accuracy in identifying crucial gait events but also showcases its applicability and adaptability across dynamic and varying scenarios, being responsive to diverse gait events.
- Innovative weighted sleep time approach: We introduce an innovative weighted sleep time approach, which, by dynamically modulating the algorithm’s sensitivity and dormancy period, significantly enhances the accuracy and adaptability in detecting gait events.
- Adaptive threshold decision-making: We have developed an adaptive threshold decision-making rule aimed at real-time adjustment of detection thresholds for gait events. This rule is particularly effective in adapting to changes in the amplitude of gait curves across various scenarios, thus substantially improving the overall performance and adaptability in gait event detection.

## 2. Method

The DGEI algorithm, grounded in time-series analysis [26] and signal processing principles [27], employs real-time differential and integral operations to discern characteristic features within gait time series from constrained local data, facilitating the identification of pivotal gait events.

**Figure 1:**
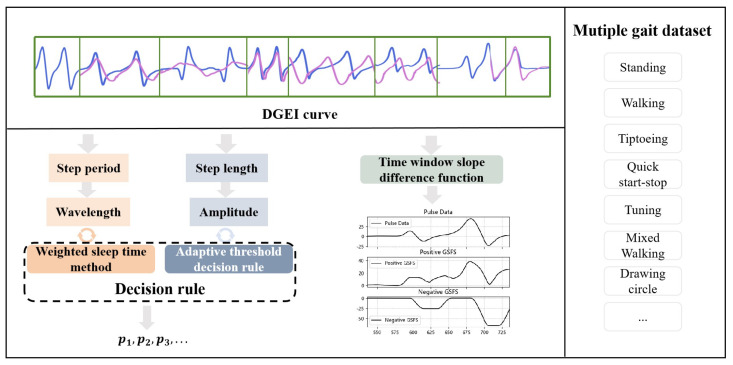
Schematic diagram of the principle of DGEI algorithm. The DGEI algorithm is able to identify and analyze various gait datasets, including standing, walking, fast start and stop, turning and mixed walking, among others.

Drawing inspiration from dynamic systems theory, this approach hinges on the identification and interpretation of patterns and trends within continuous data streams. As depicted in Figure 1, the DGEI algorithm encompasses a time-window slope differentiation function, an adaptive threshold decision rule, and a weighted sleep time method. These components collectively enable nuanced analysis and accurate recognition of gait events, reflecting the algorithm’s sophistication and adaptability in dynamic gait analysis.

### 2.1. Dynamic Gait Event Identifier

The DGEI algorithm distinctly emphasizes local amplitude variations within data to identify specific gait cycle events, such as heel strike (HS) [28] and toe-off (TO) [29]. DGEI’s objective is to delineate the ascending or descending slopes of the gait curve via sequential frame analysis within a sliding time window, culminating in the formulation of the DGEI curve. To facilitate the detection of peaks and troughs in the gait curve, DGEI incorporates $DGEI_{pos}$ and $DGEI_{neg}$ methodologies, respectively defined as follows:

(1) $$DGEI_{pos}=\sum_{k=i-w}^{i}\left(\alpha \Delta y_{k}+\beta \frac{\Delta y_{k}}{\Delta t_{k}}\right)\cdot 1_{\Delta y_{k}>Bar}$$

(2) $$DGEI_{neg}=\sum_{k=i-w}^{i}\left(\alpha \Delta y_{k}+\beta \frac{\Delta y_{k}}{\Delta t_{k}}\right)\cdot 1_{\Delta y_{k}\leq Bar}$$

where $1_{\Delta y_{k}>Bar}$ is the indicator function, i.e.,

(3) $$1_{\Delta y_{k}>\mathrm{Bar}}=\left\{\begin{array}{cc} 1, & \Delta y_{k}>\mathrm{Bar}\\ 0, & \Delta y_{k}\leq \mathrm{Bar} \end{array}\right.$$

Wherein:

- $\Delta y_{k}=y_{k}-y_{k-1}$ represents the change in Euler angles between consecutive gait data points, signifying the instantaneous gait dynamics.
- $\Delta t_{k}$ denotes the time interval between these data points, reflecting the temporal aspect of gait changes.
- α and β are weighting coefficients designed to balance the immediate gait changes against their rate over time, thereby accommodating the diverse dynamics of gait patterns. These coefficients are defined as follows:
  (4) $$\alpha =\frac{\sigma_{\Delta y}}{\sigma_{\Delta y}+\mu_{\Delta v}}$$
  (5) $$\beta =\frac{\mu_{\Delta v}}{\sigma_{\Delta y}+\mu_{\Delta v}}$$
- $\sigma_{\Delta y}$ is the standard deviation of the immediate changes in Euler angles ($\Delta y_{k}$), a statistical measure capturing the variability within the gait data.
- $\mu_{\Delta v}$ represents the mean rate of change in the Euler angles, encapsulating the average velocity of gait alterations across the dataset.
- Bar sets the threshold, distinguishing between positive and negative gait events in the context of the algorithm’s classification process.

**Figure 2:**
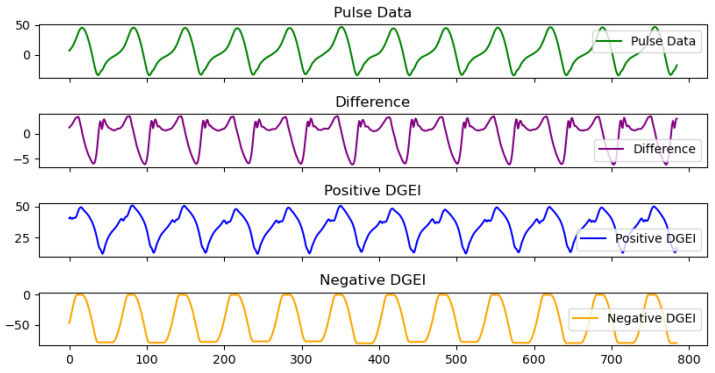
A detailed view of the gait analysis facilitated by the Dynamic Gait Event Identifier (DGEI).

To elucidate the practical application and efficacy of the DGEI algorithm, we present a series in Figure 2 that visually depicts the relationship between raw pulse data, differential signals, and the resultant DGEI outputs. DGEI operates by first extracting the first-order difference of the pulse data, representing the gait signal’s moment-to-moment variations. This differential signal is pivotal, serving as the foundation upon which the DGEI constructs its analysis. The green curve in the first row corresponds to the purple curve in the second row. Similarly, the value corresponding to the integral above zero in the time window of the purple curve is the blue curve, positive DGEI, in the third row. The values corresponding to the integrals below zero in the time window are the values of the orange curve in the fourth row.

The algorithm then aggregates these categorized changes over a designated time window, yielding two distinct signals: positive DGEI and negative DGEI. The positive DGEI signal captures the accumulation of upward trends in the gait cycle, highlighting phases of increased activity or movement intensity. Conversely, the negative DGEI signal reflects the accumulation of downward trends, providing insight into periods of reduced activity or deceleration.

In this illustration, we observe the layered approach of DGEI in action. The topmost graph showcases the raw ’pulse data’, with identifiable peaks corresponding to key moments within the gait cycle. The subsequent graph reveals the differential signal derived from this pulse data, emphasizing the rate of change across the gait cycle. The final panels vividly display the ’positive DGEI’ and ’negative DGEI’ signals, respectively, offering a clear and intuitive understanding of the upward and downward trends that characterize human gait.

**Figure 3:**
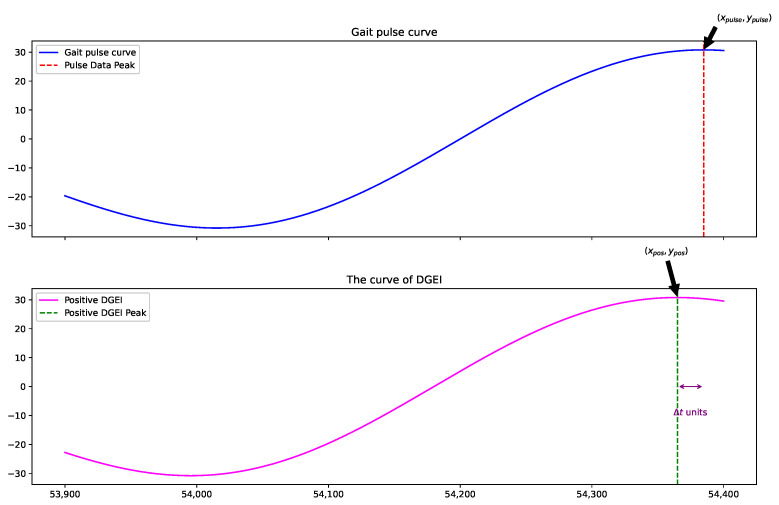
Advance identification of gait cycle peak points. The red dashed line represents the peak of the “pulse data” curve and the green represents the “DGEI” curve. The purple double-headed arrows, labeled Δt, indicate the number of frames predicted in advance.

DGEI can also be used for prereaction in gait event recognition. This anticipatory capability stems from the DGEI curve’s analytical focus on the rate of change in the gait data. When the gait curve’s upward trajectory starts to slow, indicating a forthcoming peak, the DGEI curve reacts to this deceleration, as shown in Figure 3.

The inherent sensitivity of the DGEI curve to changes in slope is the cornerstone of its capability to preemptively recognize peaks within the gait cycle. This sensitivity ensures that as the gait curve approaches a peak—a point where the slope begins to decline—the DGEI algorithm can detect this transition ahead of time. Essentially, the algorithm is attuned to the rate at which gait characteristics change, not just the changes themselves. As the gait curve’s slope increases, indicating an impending peak, the DGEI curve accumulates these positive changes, effectively ‘predicting’ the peak before the actual slope decrease occurs.

The curve of DGEI can be observed to identify the peak point Δt frames in front of the “pulse data”. This early detection allows for faster response and prediction of critical events during the gait cycle, which is essential for scenarios that require fast and accurate responses, such as dynamic balance control, person recognition, and motion analysis.

In practical gait analysis, pulse peak formation manifests in two distinct scenarios: peaks initiated by toe-off events and those arising from noise due to foot tremors. It is imperative to distinguish between these, as only the former scenario yields valid data for gait prediction. Upon peak detection, it becomes crucial to deploy decision rules that transform extracted gait curve features into corresponding gait events. These rules are categorized into three key aspects:

Firstly, within a continuous gait curve, alternating occurrences of peaks are expected, as each gait cycle typically features a single instance of both heel strike (HS) and toe-off (TO) events.

Secondly, the walking pace necessitates a temporal gap between two identical gait events. This criterion aids in differentiating effective peaks induced by toe-off from those generated by foot tremors. In actual gait analysis, following the recognition of a toe-off-induced peak, the algorithm transitions into a “sleep” phase, temporarily halting the detection of new peaks. This approach is grounded in human gait biomechanics: two toe-off events within a standard gait cycle are improbable, indicating that a subsequent peak may merely be noise.

Thirdly, in both the gait and DGEI curves, the peaks and troughs linked to TO and HS events typically represent the cycle’s maximal or minimal values. Hence, these extreme values within a cycle are not subject to detection. By setting a threshold baseline *k*, the method filters these extremes. Only peaks and troughs that emerge after crossing this baseline in the gait curve signal and DGEI signal are deemed valid for detection, thereby ensuring precision and relevance in the gait analysis process.

However, the simultaneous realization of real-time precision and accuracy in the second and third rules presents considerable challenges, especially in complex environments characterized by their dynamic and variable nature. Diverse gait contexts yield distinct curve features, such as amplitude, period, and phase shifts, rendering static thresholds and sleep durations less effective in these multifaceted real-world scenarios. To mitigate these challenges, we have developed a synergistic approach combining highly adaptive thresholds with a weighted sleep time methodology. This dual strategy is designed to adeptly navigate the intricacies of dynamic and multifarious gait environments, ensuring accuracy and responsiveness in gait analysis.

### 2.2. Weighted Sleep Time Method

To actualize this approach, we developed a weighted queue system, which adaptively modifies sleep durations in response to the present characteristics of gait data. The sleep interval is tailored to the individual’s step frequency and speed, varying in different walking contexts. For example, in scenarios of rapid walking, the sleep duration is curtailed to align with increased stepping rates. In contrast, during slow-paced walking, the sleep interval is extended to mitigate the impact of noise. The data are only admitted into the pulse peak window queue when they align with the predefined time intervals specific to the gait scenario. This method effectively sieves out noise while simultaneously ensuring the accurate capture of critical gait events. The mathematical formulation of the weighted sleep time approach is delineated as follows:

(6) $${\mathrm{Sleeptime}}^{\left(n\right)}=\frac{\sum_{i=1}^{q}RT_{i}^{\left(n\right)}\times w_{i}}{\sum_{i=1}^{q}w_{i}}$$

In our model, we consider a peak time window queue denoted as *Q*, wherein ${Sleeptime}^{\left(n\right)}$ corresponds to the minimal sleep duration for the *n*th significant peak. The length of the queue is represented by *q*, and $RT_{i}^{\left(n\right)}$ signifies the sleep duration recorded at the *i*th position within *q* for the calculation of the *n*th significant peak. Additionally, $w_{i}$ illustrates the weight assigned to the *i*th position in the queue. Our framework asserts that weights are more substantial closer to the queue’s entrance. Therefore, from the queue exit to the entrance, $w_{i}$ incrementally increases from 1 to *q*.

### 2.3. Adaptive Threshold Decision Rules

In confronting the diverse amplitude alterations in gait curves encountered across various scenarios, our research introduces an innovative adaptive threshold decision rule. This rule is designed for the instantaneous recalibration of the detection threshold for pulse peaks, thereby elevating the accuracy and adaptability of peak identification.

Rooted in a straightforward yet efficacious observation, the adaptive threshold decision rule acknowledges that gait curve amplitudes differ across distinct scenarios. Activities characterized by rapidity or intensity are likely to result in more pronounced amplitudes, in contrast to the subdued amplitudes observed in slower or more moderate activities. Consequently, employing a static threshold for pulse peak detection proves inadequate for encompassing the entire spectrum of scenarios. The algorithm, therefore, necessitates a real-time modulation of the pulse peak detection threshold, accommodating the variability in individual gait patterns and environmental dynamics.

To tackle this challenge, our research has formulated a dynamic threshold computation mechanism, diligently monitoring the pulse peaks within each position of the weighted queue in real time. For every emergent pulse peak, its threshold is established as 60% of the aggregated weighted average of existing pulse peaks within the queue. This criterion ensures that a new pulse peak is recognized as valid and allowed entry into the pulse peak queue only if it exceeds this dynamically adjusted threshold. The baseline threshold for pulse peaks is designated as Threshold0. In instances where n−1 peaks have already been validated and incorporated into the peak time window queue, the adaptive threshold for the subsequent, or *i*th, peak is determined by the following formula:

(7) $${\mathrm{Threshold}}^{\left(n\right)}=\frac{\sum_{i=1}^{q}{\mathrm{peak}}_{i}^{\left(n\right)}\times w_{i}}{\sum_{i=1}^{q}w_{i}}$$

In this context, ${\mathrm{Threshold}}^{\left(n\right)}$ refers to the threshold for the *n*th validated peak, whereas ${\mathrm{peak}}_{i}^{\left(n\right)}$ denotes the peak value located at position *i* within the queue *Q* for the calculation of the *n*th threshold of the pulse peak. This formulation is integral to our dynamic threshold computation mechanism, ensuring the precision of peak validation in the analysis of gait data.

## 3. Experiment

### 3.1. Data Gathering

In this exploratory study, we engaged four healthy male subjects (age: 23.3 ± 1.7 years; weight: 60.3 ± 22.5 kg; height: 179 ± 6.2 cm), none of whom had any notable physical or cognitive impairments that could influence their gait. Participants were outfitted with a singular inertial measurement unit (IMU) (XSens DOT, Enschede, The Netherlands), securely positioned on the anterior section of the tibia using an adjustable nylon buckle strap. The IMU maintained connectivity with a handheld device via Bluetooth, facilitating data transmission at a frequency of 60 Hz. Precisely aligned along the longitudinal axis of the tibia, the IMU was calibrated to accurately capture the tibial angular velocity in the sagittal plane, focusing primarily on the *x*-axis.

In an endeavor to encompass the dynamic spectrum of human gait, our study specifically targeted two principal gait modalities: natural and pathological. Natural gait entailed extended periods of consistent ambulation interspersed with phases of rest, including rapid transitional movements, turns, and static standing phases [30]. The primary objective for the algorithm was to achieve optimal stability and precision. Pathological gait, on the other hand, was characterized by increased tremors and erratic fluctuations in frequency and amplitude. Data acquisition spanned across these varied states, capturing transitions from stillness to motion and vice versa for each participant. The comprehensive dataset encompassed around 1500 individual gait samples, divided into several distinct walking patterns including regular, rapid transitions, turning, standing, shuffling, and specific pathological gaits like simulated Parkinsonian movements. The IMU gathered crucial data encompassing tibial angular velocity, acceleration, and step timing, with gait events (HS, TO, WS, WP) meticulously annotated through video assessment and non-real-time processing. Moreover, all data compilation was executed within a regulated setting, employing splicing techniques for real-time algorithmic detection, thereby simulating authentic scenarios of varied walking patterns interspersed with stationary periods, thus ensuring the fidelity and dependability of our findings.

### 3.2. Evaluation Indicator

To accurately assess the performance of the proposed algorithm, we utilized three key metrics: sensitivity, average difference, and the Matthews correlation coefficient (MCC). These metrics provide a comprehensive understanding of the algorithm’s effectiveness in practical applications.

1. Sensitivity: Also known as the true positive rate, this metric measures the proportion of correctly identified true positives (e.g., accurately detected gait events) to the total actual positives. It is a crucial indicator of the algorithm’s capability to capture all relevant events. The formula for sensitivity is as follows:

(8) $$Sensitivity\left(\%\right)=\frac{TP}{TP+FN}$$

where TP is the number of true positives, and FN is the number of false negatives.

2. Average difference: This metric measures the mean absolute difference between the positions of events detected by the algorithm and the actual event positions. It reflects the algorithm’s precision in event localization and is an important standard for assessing accuracy. The formula for average difference is

(9) $$Average\;Difference=\frac{1}{N}\sum_{n=1}^{N}\left|P_{actual}\left(n\right)-P_{detected}\left(n\right)\right|$$

where *N* is the total number of events, $P_{actual}\left(n\right)$ is the position of the *n*th actual event, and $P_{detected}\left(n\right)$ is the position of the *n*th detected event.

3. Matthews correlation coefficient (MCC): The MCC is a value between −1 and 1 that measures the quality of binary (two-class) classifications. It takes into account all four values of the confusion matrix (TP, FP, TN, FN), providing a balanced measure that offers useful information even in situations of class imbalance. The formula for MCC is

(10) $$MCC=\frac{\left(TP\times TN)-(FP\times FN\right)}{\sqrt{\left(TP+FP)(TP+FN)(TN+FP)(TN+FN\right)}}$$

An MCC value closer to 1 indicates better performance of the algorithm; a value closer to −1 indicates poorer performance; and a value close to 0 indicates that the performance of the algorithm is no better than random guessing.

### 3.3. Optimization Methodology

Hyperparameter optimization [31] plays a critical role in enhancing the performance and efficacy of machine learning algorithms by systematically searching for the ideal set of hyperparameters. In the context of our Dynamic Gait Event Identifier (DGEI) methodology, two significant hyperparameters, *sleeptime* and *bar*, are paramount to optimizing the algorithm’s capability to accurately detect and analyze gait events in real time.

Grid search [32], widely used in hyperparameter optimization for machine learning models [33] and sophisticated algorithms [34], can systematically evaluate all possible combinations of hyperparameters, ensuring the best set is identified for model performance optimization.

Our approach to hyperparameter optimization involved a comprehensive grid search across a predefined range of values for *sleeptime* (0–100) and *bar* (0–30), with an increment step of 1. This systematic exploration was aimed at identifying the optimal settings that enhance the DGEI algorithm’s performance, specifically focusing on sensitivity, average difference, and the Matthews correlation coefficient (MCC) as key metrics.

**Figure 4:**
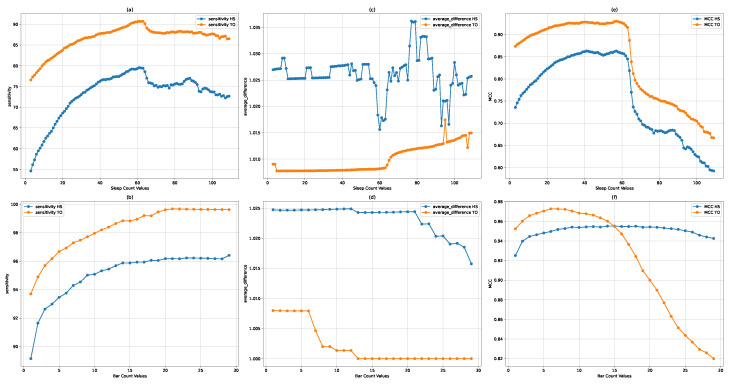
Comprehensive visualization of hyperparameter optimization results for the DGEI methodology: The top row (**a**,**c**,**e**) presents the optimization scenarios for the ‘sleeptime’ hyperparameter. The bottom row (**b**,**d**,**f**) illustrates the optimization outcomes for the ‘bar’ hyperparameter.

Through our grid search analysis, we generated Figure 4 depicting the performance of each hyperparameter setting against the sensitivity, average difference, and MCC metrics. This visual representation enabled a clear identification of the optimal hyperparameter values that maximize the DGEI algorithm’s efficacy.

#### 3.3.1. Optimization of Sleeptime

The optimization of the *sleeptime* hyperparameter was conducted with a focus on three crucial performance metrics: sensitivity, average difference, and the Matthews correlation coefficient (MCC). Our analysis revealed distinct patterns of performance variation across these metrics as *sleeptime* values were adjusted in Figure 4a,c,e.

- Sensitivity analysis: The sensitivity metric, which indicates the true positive rate of detecting gait events, showed peak values for both toe-off (TO) and heel strike (HS) events in the range of 60 to 63 s. This suggests an optimal balance between event detection capability and the algorithm’s responsiveness within this *sleeptime* interval.
- Average difference optimization: The average difference, reflecting the precision in localizing detected events, reached its optimum at a *sleeptime* of 60 s. This optimal point signifies the highest alignment between detected events and their actual occurrences, thereby minimizing localization error.
- MCC performance: The Matthews correlation coefficient, a comprehensive measure of classification accuracy, exhibited optimal performance within the 55 to 60 s range. Given the MCC’s value in assessing the balance between various aspects of binary classification performance, this finding underscores the efficacy of the DGEI methodology within the specified *sleeptime* range.

Given these observations, a *sleeptime* of 60 s was selected as the optimal setting, harmonizing the algorithm’s sensitivity, precision, and overall classification accuracy.

#### 3.3.2. Optimization of Bar

The *bar* hyperparameter’s optimization was similarly assessed across the same metrics in Figure 4b,d,e, revealing the following insights:

- MCC considerations: The MCC metric, which offers a balanced evaluation of the algorithm’s classification capabilities, identified the 5 to 10 range as optimal. This interval demonstrates a robust performance across detecting true positives and negatives while minimizing errors.
- Sensitivity insights: For the sensitivity metric, values above 8 consistently achieved a performance exceeding 95%. This high sensitivity indicates the algorithm’s effective detection of gait events at higher *bar* settings.
- Average difference analysis: The average difference across varying *bar* values showed minimal variation, suggesting that this metric was less sensitive to changes in the *bar* parameter. This stability implies that the *bar* setting’s impact on event localization precision is comparatively uniform.

Considering the comprehensive analysis across all metrics, a *bar* value of 9 was determined to be optimal, striking an effective balance between sensitivity, specificity, and overall classification accuracy.

The application of the optimized hyperparameters markedly improved the DGEI methodology’s detection accuracy and precision. With *sleeptime* set at 60 and *bar* at 6, the algorithm not only achieved a near-perfect alignment with ground truth data but also showcased its robustness in varying gait conditions.

#### 3.3.3. Coupling Analysis of Hyperparameters

In the context of optimizing the Dynamic Gait Event Identifier (DGEI) methodology, it was crucial to evaluate the interdependence or coupling between critical hyperparameters to ensure their independent optimization did not detrimentally impact the algorithm’s overall performance.

To assess the coupling effect between *sleeptime* and *bar*, we employed a three-dimensional visualization approach. This method involved plotting the performance metrics—sensitivity and Matthews correlation coefficient (MCC)—against both hyperparameters on a three-dimensional graph. The aim was to observe the contours and surfaces formed by varying both parameters simultaneously, which could indicate areas of optimal performance and potential interaction effects between the parameters.

**Figure 5:**
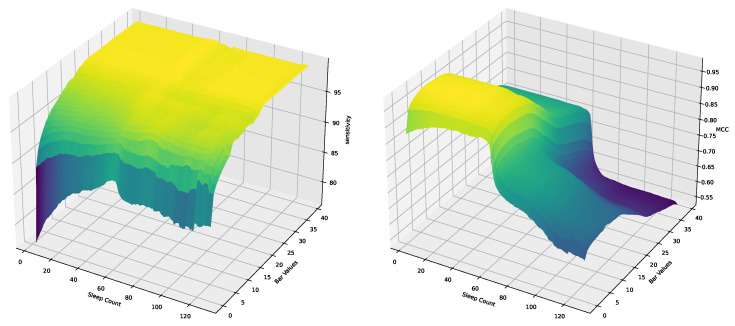
The 3D coupling of sensitivity and MCC with two hyperparameters.

Figure 5 revealed several key insights into the interactions:

1. Surface analysis: The plots showed smooth surfaces without abrupt changes or discontinuities, suggesting that the interaction between *sleeptime* and *bar* does not lead to sudden performance degradations within the explored ranges.
2. Optimal region identification: A region of the surface consistently demonstrated superior performance across all metrics, located around *sleeptime* = 60 and *bar* = 9. This observation confirms the previously determined optimal settings but also emphasizes their robustness in conjunction with one another.
3. Coupling effect: While varying one hyperparameter affected the performance metrics, the changes were gradual and predictable, indicating a low coupling effect.

The coupling analysis decisively showed that *sleeptime* and *bar* could be optimized independently without adversely affecting the DGEI methodology’s performance.

### 3.4. Peak Detection

For the dataset that includes a variety of complex scenarios following data splicing, we initially detect the WS (walking start) and WP (walking pause) events, and all such events can be detected. Subsequently, the detection of HS (heel strike) and TO (toe-off) events is carried out, with the results presented in Table 1 and Table 2.

A comprehensive analysis was conducted on the performance of gait detection using the Dynamic Gait Event Identifier (DGEI) methodology, with an emphasis on its accuracy in identifying gait events. The evaluation focused on the proximity of the DGEI algorithm’s outputs to actual values, particularly within a threshold of approximately five frames, using index-matching rules. A detailed analysis follows.

1. For the normal gait mode, including scenarios like “Sequential Ambulation + Standard Locomotion”, “Accelerated Initiation − Cessation (Universal)”, and “Standard Locomotion”, the DGEI method exhibited high accuracy. The detection rate, sensitivity, and positive predictive value (PPV) approached or reached 100%. This indicates the DGEI method’s effectiveness in identifying standard gait events.
2. In highly dynamic gait events, the scenario “Accelerated Initiation − Cessation + Rotational Movement” also nearly achieved perfect scores, demonstrating DGEI’s capability to maintain high accuracy and detection rates even in contexts with rapid changes in gait patterns.
3. Moreover, in the concatenated data, all “walking start” (WS) and “walking pause” (WP) events were identified and detected, indicating DGEI’s clear discernment of gait cessation and initiation. Tremors during stationary phases were effectively screened and ignored by WS and WP events, explaining the high accuracy in detection.
4. For the “Static Posture” activity, a detection rate of zero was as expected, since no gait activity occurs in this scenario, thus no heel strike (HS) or toe-off (TO) events are produced. This result confirms DGEI’s specificity, as it correctly does not misidentify stationary states as gait events.
5. In abnormal gait modes, such as “Dragging” and “Extension Beyond Normal Limits”, the DGEI method showed some reduction in sensitivity. Particularly in the “Dragging” activity, despite a still high detection rate, sensitivity dropped to 68.75%. This decrease could be attributed to the irregularities in the gait pattern affecting the performance of the DGEI method, leading to reduced detection efficacy.
6. In pathological gait simulations such as “Flexural Rigidity” and “Neurological Disorder Gait”, the DGEI method still demonstrated relatively high detection rates and sensitivity, indicating its applicability for certain pathological gait events.
7. The DGEI method exhibited very low mean absolute difference (MAD) values in most gait activities, particularly in scenarios like “Sequential Ambulation + Standard Locomotion”, “Accelerated Initiation − Cessation (Universal)”, and “Standard Locomotion”, where the MAD was almost zero. This implies that there was no deviation between the time points generated by the algorithm and those manually annotated, indicating very high accuracy.
8. For the “Dragging” activity, the MAD value significantly increased to 3.32 samples. This is an important indicator that the algorithm might struggle to accurately identify gait events in irregular gait patterns, leading to larger time discrepancies.

### 3.5. Error Analysis

**Figure 6:**
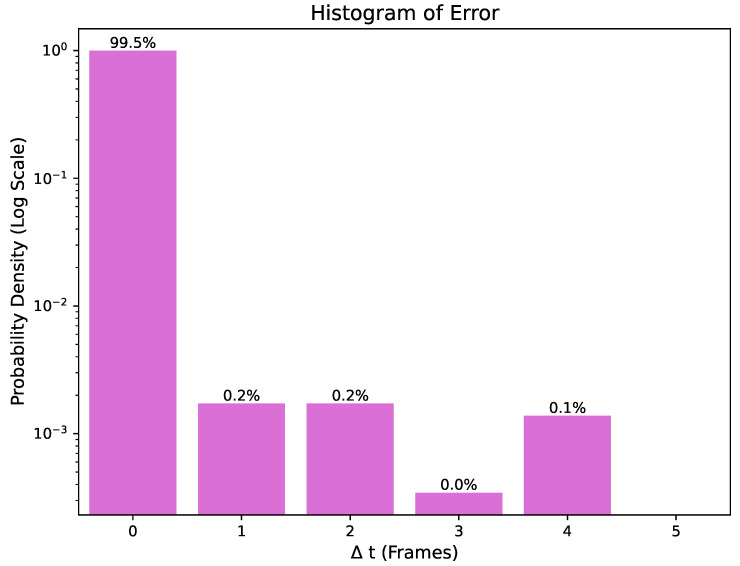
The error distribution between the time frame of the matching event and the ground truth is compared with the normal distribution.

In the error analysis, the detected heel strike (HS) and toe-off (TO) events showed high accuracy in terms of timing. Among them, 99.2% of the detected events match the actual occurrence time exactly, and the time difference is 0. The experimental results show that, in most cases, the adopted detection algorithm can predict gait events with high accuracy. Only 0.8% of the remaining events show slight temporal differences, as shown in Figure 6. These differences are in the range of three frames and follow a normal distribution. Such errors may arise from dynamic oscillations during gait or from the inherent irregularity and uncertainty features of pathological gait patterns.

In pathological gaits, non-linear and non-standardized gait patterns can increase the difficulty of detection. For example, patients with neurological disorders may exhibit varying gait rhythms and strides at different times, impacting the precision of the algorithm. The high dynamics and uncertainties of these pathological gaits are key factors leading to errors in the detection algorithm in a minority of cases.

It is noteworthy that, despite these errors, inaccurately detected events are generally associated with a larger amount of abnormal data. This suggests that the algorithm can make highly accurate predictions for the vast majority of gait events under normal operation.

## 4. Discussion

The DGEI algorithm introduced in this study demonstrates a significant advancement in the field of real-time gait event detection, showcasing impressive accuracy rates in identifying key gait events such as heel strike (HS) and toe-off (TO). However, to solidify its standing and ensure its broader applicability, a comparative analysis with existing methodologies is warranted.

Existing gait analysis methods vary in their approach, from video-based motion capture systems [35] that offer high spatial resolution but require controlled environments, to force plate systems [36] that provide precise measurements yet are limited by their stationary nature. Wearable sensors, including IMUs, have gained popularity due to their portability and ability to collect data in naturalistic settings [1], but their performance can be inconsistent due to sensor noise and the need for frequent calibration.

In contrast, the DGEI algorithm’s strength lies in its dynamic feature extraction strategies and real-time data analysis capabilities, which are particularly tailored for embedded system constraints. The innovative use of first-order differential integration and adaptive thresholding allows for a more nuanced understanding of gait dynamics, which sets it apart from traditional methods that often rely on fixed thresholding and may not adapt well to varying gait conditions.

Future research should aim to expand the subject pool and include a wider range of gait patterns, such as those from patients with neurological disorders or amputees using prosthetic limbs. Additionally, comparative studies with other state-of-the-art algorithms will provide insights into the DGEI’s relative advantages and potential areas for improvement. Such comparisons should consider not only accuracy metrics but also computational efficiency, ease of use, and the ability to adapt to different environmental conditions.

## 5. Conclusions

This study introduces the Dynamic Gait Event Identifier (DGEI) algorithm, setting a new standard for precision in the real-time detection of gait events across a diverse range of patterns. Through meticulous optimization, “sleeptime” and “bar” were identified as optimal at 60 and 9, respectively, with analyses confirming these settings and revealing no significant coupling effects. Demonstrating exceptional accuracy and adaptability, the DGEI algorithm excels in dynamic scenarios and accurately distinguishes between genuine gait events and noise, despite identified limitations in detecting certain abnormal gait patterns. Future efforts will aim to refine DGEI’s performance further and validate its efficacy across various populations. This work lays a foundational step toward advanced gait analysis, promising significant implications for clinical diagnostics and rehabilitation.

## Figures and Tables

**Table 1:** The assessment results of HS.

Gait Variation	Sample Size	Detection Rate	Average Deviation	Sensitivity
Sequential Ambulation + Standard Locomotion	554	100.00%	0.00	99.46%
Standard Locomotion + Rotational Movement	440	99.32%	0.15	95.21%
Accelerated Initiation − Cessation + Rotational Movement	109	92.66%	0.00	82.79%
Accelerated Initiation − Cessation (Universal)	51	100.00%	0.00	89.47%
Standard Locomotion	31	100.00%	0.00	96.88%
Static Posture	53	0.00%	0.00	0.00%
Flexural Rigidity	0	94.55%	11.81	96.30%
Floor Cleaning Activity	55	78.79%	0.96	85.25%
Circular Drawing Motion	66	100.00%	1.41	95.92%
Digitigrade Locomotion	47	100.00%	0.00	98.00%
Neurological Disorder Gait	58	100.00%	0.00	95.08%
Extension Beyond Normal Limits	49	90.57%	1.38	87.27%
Overall	1513	97.82%	0.58	94.33%

**Table 2:** The assessment results of TO.

Gait Variation	Sample Size	Detection Rate	Average Deviation	Sensitivity
Sequential Ambulation + Standard Locomotion	556	100.00%	0.00	99.82%
Standard Locomotion + Rotational Movement	457	99.12%	0.22	98.26%
Accelerated Initiation − Cessation + Rotational Movement	121	100.00%	0.00	99.18%
Accelerated Initiation − Cessation (Universal)	57	100.00%	0.00	100.00%
Standard Locomotion	33	100.00%	0.00	100.00%
Static Posture	0	0.00%	0.00	0.00%
Flexural Rigidity	53	94.34%	0.00	98.04%
Floor Cleaning Activity	62	88.71%	3.32	68.75%
Circular Drawing Motion	48	100.00%	0.00	100.00%
Digitigrade Locomotion	49	100.00%	0.00	98.00%
Neurological Disorder Gait	61	98.36%	0.00	100.00%
Extension Beyond Normal Limits	53	100.00%	0.00	85.48%
Overall	1550	99.03%	0.18	96.91%

## Data Availability

The original contributions presented in the study are included in the article, further inquiries can be directed to the corresponding authors.
